# Chronic optogenetic stimulation of dentate gyrus granule cells in mouse organotypic slice cultures synaptically drives mossy cell degeneration

**DOI:** 10.1111/epi.18314

**Published:** 2025-02-12

**Authors:** Carolin Christina Koretz, Rebecca Schneider, Tassilo Jungenitz, Alexander Drakew, Jochen Roeper, Thomas Deller

**Affiliations:** ^1^ Institute for Clinical Neuroanatomy, Dr. Senckenberg Anatomy, Fachbereich Medizin Goethe University Frankfurt Frankfurt am Main Germany; ^2^ Institute of Neurophysiology, Fachbereich Medizin Goethe University Frankfurt Frankfurt am Main Germany

**Keywords:** channelrhodopsin‐2, excitotoxicity, patch‐clamp, retigabine, temporal lobe epilepsy

## Abstract

**Objective:**

Degeneration of hilar mossy cells in the dentate gyrus is an important hallmark of hippocampal sclerosis and is often observed in patients with temporal lobe epilepsy. To understand the pathogenesis of hippocampal sclerosis and develop novel neuroprotective treatments, it is critical to determine the mechanistic processes of mossy cell degeneration and factors that influence cell vulnerability or resilience. However, suitable in vitro approaches are currently lacking. We have developed and validated an organotypic slice culture‐based in vitro model that facilitates mechanistic studies of activity‐dependent mossy cell vulnerability and resilience.

**Methods:**

A model was developed using entorhino‐hippocampal slice cultures. Dentate gyrus granule cells were transduced with adeno‐associated viruses to express channelrhodopsin2. Transduced cultures were chronically stimulated by light, and resulting cell damage was assessed by propidium iodide staining. Spontaneous synaptic activity before and after optical stimulation was recorded using whole‐cell patch‐clamp.

**Results:**

Selective and dose‐dependent hilar neuron degeneration was observed following chronic optogenetic stimulation of organotypic slice cultures expressing channelrhodopsin‐2 in granule cells. Treatment with the anticonvulsant retigabine reduced stimulation‐induced hilar neuron loss in a dose‐dependent manner. This demonstrates the suitability of our optogenetic in vitro model for drug screening. Patch‐clamp recordings verified strong synaptic activation of mossy cells during optical stimulation and a reduction in spontaneous excitatory synaptic activity after stimulation.

**Significance:**

The role of mossy cells in the context of epileptic seizures has been a controversial topic of discussion. The presented in vitro model allows the study of mossy cell vulnerability on a single‐cell level and provides the first evidence for changes in synaptic activity after stimulation. This model will facilitate our mechanistic understanding of temporal lobe epilepsy, providing a foundation for novel therapeutic interventions aimed at preserving mossy cell function in epilepsy patients.

**Key points**

- Optogenetic stimulation of dentate gyrus granule cells leads to strong synaptic activation of mossy cells.
- Optogenetic stimulation of granule cells leads to selective damage of hilar neurons.
- Optogenetic stimulation in organotypic slice cultures can be used to evaluate the protective effects of drugs on mossy cells.
- After optogenetic stimulation, mossy cells exhibit a significant decrease in spontaneous excitatory postsynaptic potential frequency and show signs of dendritic pathology.

## 1 INTRODUCTION

Mossy cells (MCs), located in the hilar region of the dentate gyrus (DG), are increasingly recognized for their important role in spatial memory encoding in the hippocampus.^1^ These glutamatergic neurons are distinguished by their large somata situated in the hilus, and by their proximal dendrites exhibiting large, complex spines.^2^, ^3^ Electrophysiologically, MCs are characterized by their regular spiking properties and show a high frequency of excitatory postsynaptic potentials (EPSPs).^4^, ^5^

In the context of temporal lobe epilepsy (TLE), MCs have attracted considerable attention due to their vulnerability to damage caused by overexcitation.^6^ One of the most common histopathological findings in patients with TLE is hippocampal sclerosis, characterized by substantial neuron loss in the hippocampus, including the hilar region.^7^, ^8^ Specifically, in a pathological subtype known as hippocampal sclerosis type 3 or end folium sclerosis, significant neuronal loss occurs in the hilus, whereas CA3, CA2, and CA1 pyramidal cell populations are less affected.^9^ It has been debated whether this selective cell loss disrupts hippocampal excitation/inhibition balance, thereby altering seizure susceptibility.^1^, ^10^, ^11^ One aspect of this debate centers on the dual role of MCs: they can directly excite granule cells (GCs) via projections onto GC dendrites in the inner molecular layer, and they can also indirectly inhibit GCs through the innervation of inhibitory basket cells.^12^ A substantial loss of hilar neurons reduces GC inhibition, leading to spontaneous GC discharges due to insufficient shunting inhibition.^13^ In vivo chemogenic silencing experiments and slice recordings while inducing spontaneous seizure‐like activity have implicated MCs in the initiation of status epilepticus.^11^ Long‐term potentiation through repeated activity at the MC–GC synapse, but not the MC–basket cell synapse, has been shown to lead to a shift in the excitation/inhibition balance, potentially contributing to the epileptogenic process.^14^

In vivo studies utilizing electrical stimulation of the perforant path have successfully replicated the selective damage pattern of end folium sclerosis in rodents, emphasizing the hilar region's susceptibility to selective neuronal loss.^6^, ^15^, ^16^ This raises the question of why MCs in particular are more vulnerable than other cell types in the DG. Determining the factors contributing to this vulnerability and identifying ways to protect the MC population remain important issues.

As we aim to define the mechanisms underlying MC vulnerability, we have developed an in vitro model based on entorhino‐hippocampal organotypic tissue cultures (OTCs) that has enabled us to induce selective hilar cell loss in the DG via specific optogenetic stimulation of GCs. The observed pattern of cell death mirrored those observed in in vivo models of TLE. Patch clamp recordings from MCs revealed a poststimulation decrease in the frequency of EPSPs.

## 2 MATERIALS AND METHODS

### 2.1 Animals

The generation of organotypic entorhino‐hippocampal tissue cultures from C57Bl6/J mice was performed in accordance with the German animal welfare law and had been declared to the Animal Welfare Officer of Goethe‐University, Faculty of Medicine (Wa‐2014‐35). Wild‐type (WT) mice (C57BL/6J background) were bred and housed at the animal facility of Goethe University Hospital Frankfurt. Animals were maintained on a 12‐h light/dark cycle with food and water available ad libitum.

### 2.2 Organotypic tissue cultures

OTCs 300 μm thick were prepared from WT mice of either sex at postnatal day 4–5 as previously described.^17^ See Appendix S1 for further details.

### 2.3 Viral labeling

To express channelrhosopsin2 variant H134R in DG GCs, OTCs were transduced with a recombinant adeno‐associated virus serotype 2 (AAV2) containing the gene for a channelrhodopsin‐2 (ChR2)–enhanced yellow fluorescent protein (eYFP) fusion protein under the calmodulin‐dependent protein kinase IIα promotor (AddGene #26969). The AAVs were diluted in phosphate‐buffered saline (PBS) to a viral titer of 2 × 10^12^ vg/ml. Local injections were performed under visual control at day in vitro (DIV) 2–4. See Appendix S1 for further details.

### 2.4 Optogenetic stimulation in the incubator

At 28 days post‐AAV injection, OTCs were optically stimulated within the incubator. The stimulation device consists of a box containing light‐emitting diode (LED) modules positioned underneath the OTCs to be illuminated, and a separate Raspberry Pi‐controlled LED driver located outside of the incubator. See Appendix S1 for further details.

In cases of pharmacological treatment, the substances were applied to the cell culture medium before the start of the optical stimulation: 10 μmol·L^−1^ D‐2‐amino‐5‐phosphonopentanoic acid (D‐AP5; Alomone Labs) or 10 μmol·L^−1^ 6‐cyano‐7‐nitroquinoxaline‐2,3‐dione (CNQX; Alomone Labs) dissolved in H_2_O.

### 2.5 Propidium iodide staining

Immediately after the optical stimulation, the nucleic acid‐intercalating dye propidium iodide (PI; Thermo Fisher Scientific) was added to the culture medium (final concentration = 5 μg/mL). After 2 h of PI‐incubation, OTCs were washed with PBS and fixed with 4% paraformaldehyde/4% sucrose in PBS for 1 h. Following fixation, OTCs were washed with PBS and stained with the nuclear marker TO‐PRO‐3 (Invitrogen; 1:5000 in PBS) for 10 min. After another two washes with PBS, OTCs were mounted onto glass slides using fluorescence mounting medium (Dako Omnis, Agilent).

### 2.6 Immunocytochemistry

For immunofluorescent stainings, slices were resectioned at 40 μm using a vibratome (VT1000 S, Leica) after PI staining and fixation (see above). Free‐floating slices were incubated in a blocking buffer containing .1% Triton X‐100 and 10% normal goat serum (NGS; Invitrogen) in PBS for 60 min at room temperature, followed by incubation in the primary antibody (rabbit anti‐calretinin CR7697 [Swant] diluted 1:500 in .1% Triton X‐100 and 10% NGS in PBS) for 2 days at 4°C. After 4× washing with PBS, sections were incubated with the secondary Alexa‐conjugated antibody (goat anti‐rabbit Alexa 405 [Invitrogen] diluted 1:500 in .1% Triton X‐100 and 10% NGS in PBS) for 4 h at room temperature, then washed in PBS four times, and finally mounted in fluorescence mounting medium (Dako‐Omnis, Agilent).

### 2.7 Confocal images of fixed tissue

Confocal image stacks (1024 × 1024 pixels) of fixed OTCs were acquired with a laser scanning microscope (Nikon C2si) equipped with two GaAsP detectors using NIS‐Elements software. Imaging was done using 10x or 20x objectives (.3 numerical aperture [NA] or .5 NA, Nikon) or a 60x oil‐immersion objective (1.4 NA, Nikon). Z‐stacks with the 10× objective were acquired with a step size of 5 μm, and z‐stacks with the 60x objective were acquired with a step size of .2 μm.

### 2.8 Image processing

For further analysis and image processing, only cultures with a sufficient ChR2‐eYFP expression level were selected. For evaluation of the expression level, the mean eYFP fluorescence in the DG area was measured in maximum intensity projections of the acquired z‐stacks. Image stacks were edited with ImageJ (v1.54f) as part of the Fiji distribution package.^18^ For semiautomated counting of PI‐positive cells, images were analyzed in batch mode, applying the same steps to all images: the creation of a maximum intensity projection, manual selection of the hilar area by hand using a polygon selection, the creation of a thresholded binary mask of the PI channel, and the counting of objects using the Particle Analyzer plugin. Sample images were brightness‐ and contrast‐ edited. All images were edited using the same settings irrespective of treatment group.

### 2.9 Retigabine treatment

For treatment with retigabine, OTCs were optically stimulated and subsequently stained with PI as described above. Retigabine dissolved in dimethylsulfoxide (Sigma‐Aldrich) was added to the culture medium during stimulation in increasing concentrations to obtain a dose–response curve. Following the PI staining, the OTCs were fixed and counterstained with TOPRO‐3, and confocal image stacks were acquired as described above. PI‐positive cells in the image stacks were counted manually.

Only experiments with at least 10 cultures were included in the analysis. Analyses and curve fitting were performed using GraphPad Prism v10. For the nonlinear regression curve fitting, the PI count data were log‐transformed and normalized to the untreated control data before modeling the nonlinear fit (variable slope, four parameters, least squares fit).

### 2.10 Whole‐cell patch‐clamp recordings

Whole‐cell patch‐clamp recordings of DG GCs and hilar MCs were performed 28–54 days after viral transduction. The membrane inserts with the cultures were transferred to a custom‐made recording chamber on the stage of an upright confocal microscope. Recordings were performed in artificial cerebrospinal fluid at 35°C.

Patch‐pipettes of 4–6 MΩ were pulled from borosilicate glass (GC150TF‐10, Harvard Apparatus) with a DMZ universal puller (Zeitz) and filled with K‐gluconate‐based internal solution containing 100 μmol·L^−1^ Alexa 647 dextran (Thermo Fisher Scientific).

Spontaneous and optically induced activity was recorded in current‐clamp mode using an ELC‐03X amplifier (npi electronic) in bridge mode. Spontaneous EPSPs were recorded for 20 min before and up to 20 min after optical stimulation.

For optical stimulation, an LED module (505 nm, LXML‐PE01‐0080, LUXEON Rebel LED) was mounted to a second micromanipulator. Light pulses were delivered for 30 min in trains of 100 pulses with 10‐ms pulse length at 12.5 Hz, with an intertrain interval of 52 s.

Confocal image stacks of the recorded cell were acquired 10 min after gaining whole‐cell access (640‐nm excitation laser only) and again after finishing the recording (488‐nm and 640‐nm excitation lasers; 2048 × 2048 pixel; zoom 1; voxel size = .104 × .104 × .5 μm). See Appendix S1 for further details.

### 2.11 Automated analysis of spontaneous EPSPs

Voltage traces of spontaneous activity (V[t]) were analyzed automatically without user interaction by applying an in‐house software routine written in LabVIEW 2023 (National Instruments). Traces were first denoised using a cubic spline fit, followed by a deconvolution (deconV[t] = V[t] + tau * dV[t] / dt).^19^ See Appendix S1 for further details.

### 2.12 Statistical analysis

For the statistical analysis of PI‐positive cells in the hilus, the Kruskal–Wallis test was used to examine differences among groups, followed by Dunn's multiple comparison test analyzing differences between pairs of treatment groups (GraphPad Prism v10). A significance level of *p* <.05 was set. Results are presented as mean ± SD.

Changes in EPSP parameters before and after optical stimulation within cells were analyzed using the paired Brunner–Munzel test implemented in R (R v4.3.2; R‐package “TOSTER”^20^, ^21^).

## 3 RESULTS

### 3.1 Optogenetic labeling and stimulation of DG GCs demonstrate synaptic connectivity between DGs and MCs in OTCs

**FIGURE 1:**
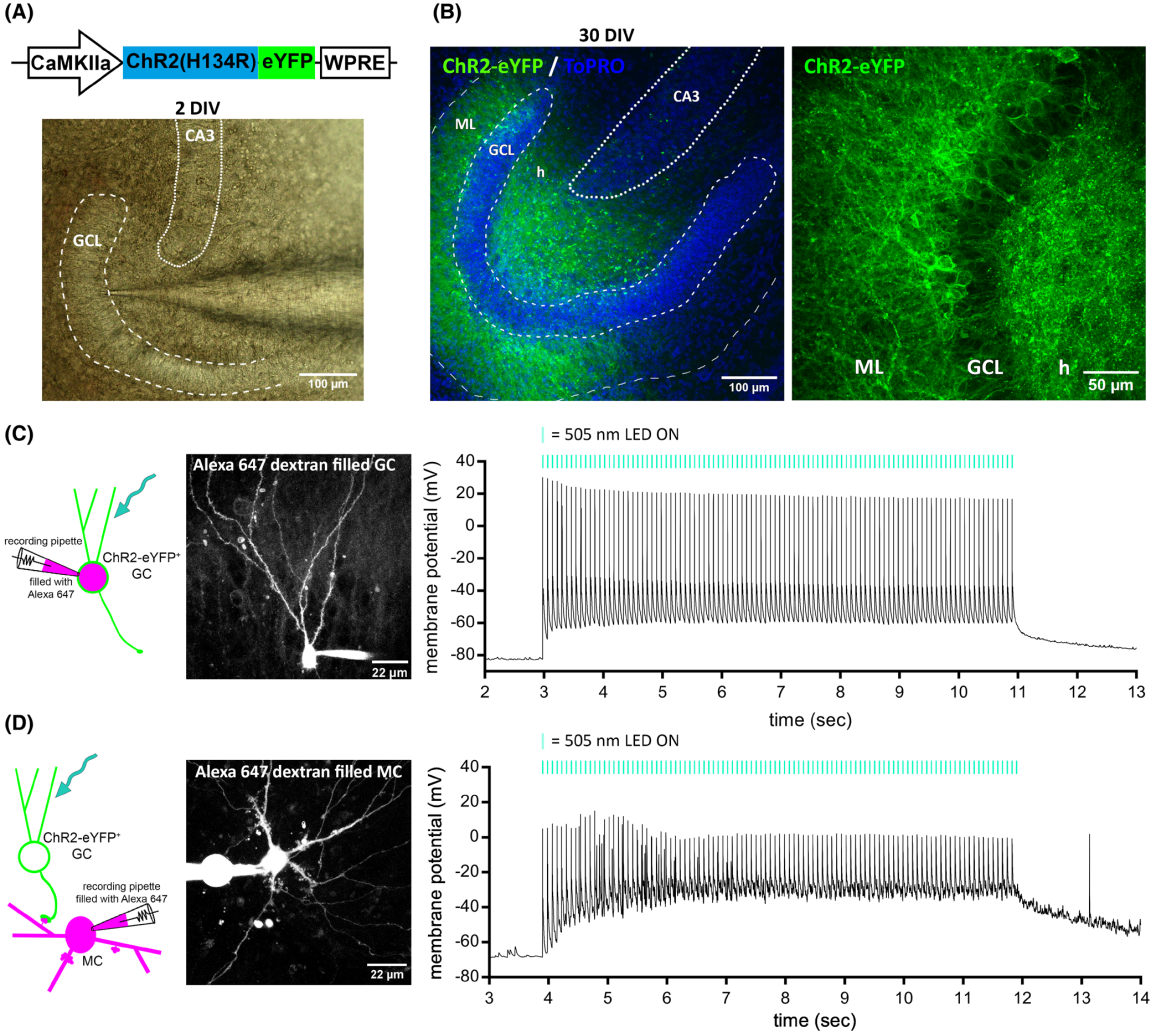
Optogenetic labeling and stimulation of dentate gyrus granule cells (GCs) show functional connectivity of GCs and mossy cells (MCs) in organotypic tissue cultures. (A) Schematic representation of the viral construct used to express the fusion protein of channelrhodopsin‐2 (ChR2) variant H134R and enhanced yellow fluorescent protein (eYFP). The construct is delivered via the visually guided local injection of adeno‐associated viruses into the GC layer (GCL) of the dentate gyrus in organotypic entorhino‐hippocampal slice cultures 2–5 days after preparation. The size of the injection was controlled using a pressure application system. Multiple injections along the GCL ensure a high density of labeled granule cells. (B) eYFP expression enables the identification of GCs expressing channelrhodopsin‐2 (ChR2). The cell layer of the dentate gyrus is visualized using TO‐PRO counterstain (blue). At 30 days in vitro (DIV), ChR2 expression is observed in the soma, dendrites, and axons of transduced GCs. The dendrites of ChR2‐expressing GCs densely label the molecular layer (ML), whereas the axons strongly label the hilar region (h). Particularly prominent are the large axon terminals, known as mossy fiber boutons, in the hilus and stratum lucidum of CA3, which display distinct ChR2‐eYFP expression. (C) Whole‐cell path‐clamp recordings demonstrate the firing of action potentials in ChR2‐expressing GCs upon optical stimulation (505‐nm light‐emitting diode [LED], 100 pulses with 10‐ms pulse length at 12.5 Hz). For direct identification of the recorded neurons, the internal solution in the patch pipette contained Alexa 647 dextran. (D) MCs, which are innervated by ChR2‐expressing GCs, exhibit strong suprathreshold synaptic activation during optical stimulation of the GCs (*n* = 17 of 17 MCs). During the 100‐pulse train, the MC shows some degree of adaptation or increased inhibition. Nevertheless, every light pulse induced a postsynaptic response in the MCs.

The in vitro system presented here enables optogenetic stimulation of DG GCs in entorhino‐hippocampal OTCs. GCs were transduced with a ChR2(H134R)‐eYFP fusion protein to render them excitable by light. Achieving a high transduction rate in GCs while ensuring specific labeling was facilitated by local, visually controlled injection of AAVs using a high‐precision pressure application system (Figure 1A). We waited until at least DIV 28 to provide enough time for the maturation of the OTCs, allowing synaptic connections to develop fully and enabling ChR2 to reach a strong level of expression. Successfully transduced GCs were identified via their eYFP expression. eYFP labeling confirmed ChR2 expression throughout the entire cell, including somatic, dendritic, and axonal compartments (Figure 1B). The spine‐bearing dendritic branches extending into the molecular layer, as well as their spreading axonal branches covering the hilar region and extending to CA3, were distinctly visible. Particularly noteworthy were the large GC presynaptic terminals in the hilar area, known as mossy fiber boutons, which exhibited robust expression of ChR2‐eYFP.

Next, we wanted to verify the light‐induced activation of ChR2‐expressing GCs. To minimize phototoxic effects during stimulation, we chose a wavelength of 505 nm, expecting approximately 60% of the maximum current response of the ChR2(H134R)‐variant.^22^ Via whole‐cell current‐clamp recordings, we validated the firing of optically evoked action potentials (APs) in ChR2‐expressing GCs stimulated with 505‐nm light pulses, which reliably entrained neuronal APs throughout the entire train of 100 light pulses at 12.5 Hz (Figure 1C). Current‐clamp recordings from identified MCs displayed robust suprathreshold synaptic activation during optical GC stimulation, indicating a strong innervation by ChR2‐expressing GCs (Figure 1D).

### 3.2 Chronic optical stimulation of GCs induces hilar neuronal cell death in the DG

**FIGURE 2:**
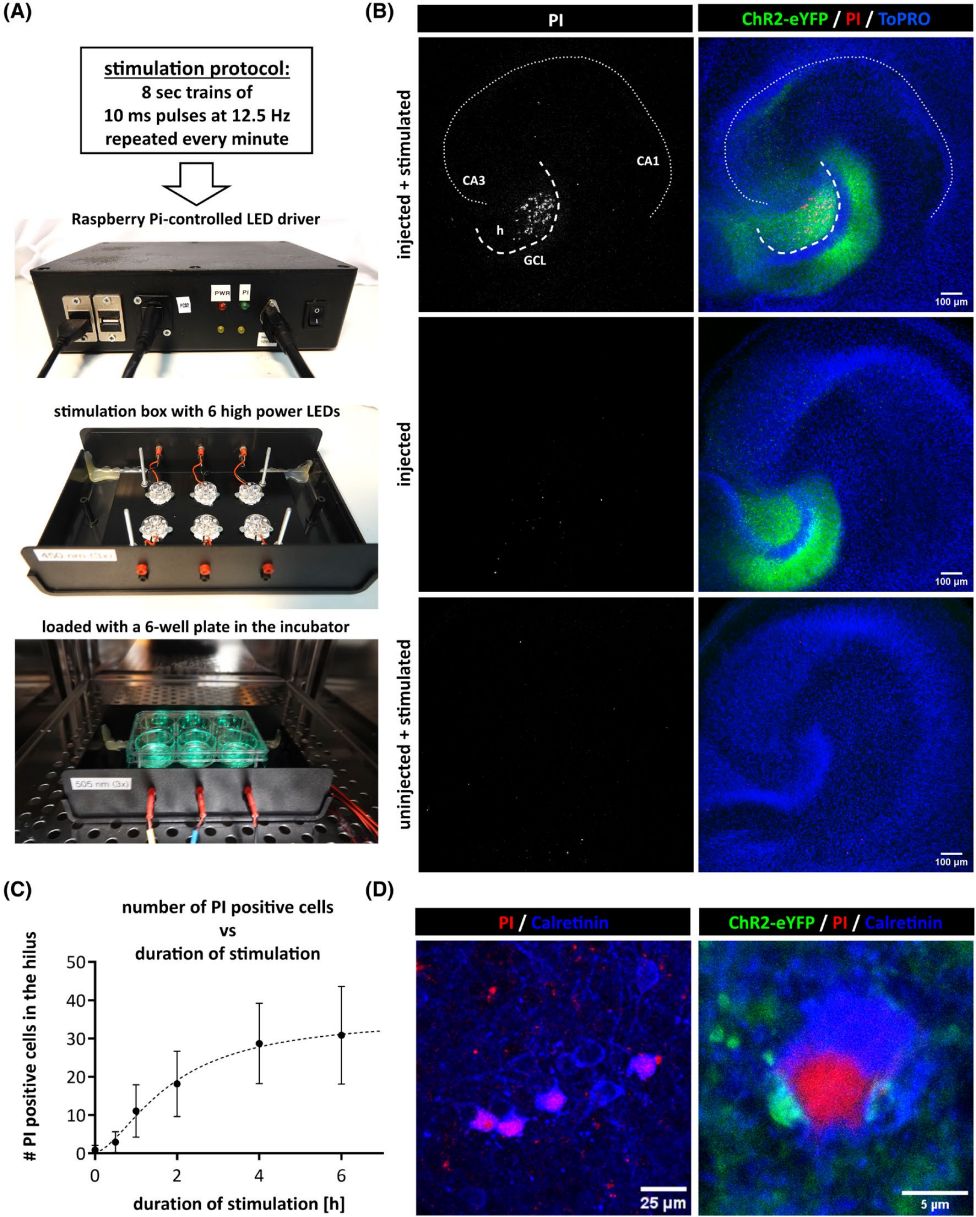
Chronic optical stimulation of granule cells induces hilar neuronal cell death in the dentate gyrus. (A) A custom‐designed stimulation device was developed for long‐term optical stimulation of organotypic tissue cultures (OTCs) within the incubator. The stimulation box is equipped with six high‐power light‐emitting diodes (LEDs) emitting 505‐nm light. OTC‐containing six‐well plates can be placed inside the box, which fits easily into the incubator. Stimulation parameters are controlled through a Raspberry Pi. (B) Optical stimulation of OTCs expressing channelrhodopsin‐2 (ChR2) over a period of 6 h resulted in cell death indicated by propidium iodide (PI) staining, specifically observed in hilar neurons of the dentate gyrus. Control OTCs, which were either ChR2‐expressing and unstimulated or ChR2‐negative and stimulated for 6 h, did not show signs of cell death. (C) Quantitative analysis of PI‐labeled cells in the hilus following repetitive stimulation protocols conducted for varying durations demonstrates a dose‐dependent effect; an increase in the number of PI‐positive cells is observed with longer stimulation periods. The dotted line represents the least‐squares fit of a sigmoidal dose–response function. *n* = 6–13 cultures per time point. (D) Immunolabeling against the calcium‐binding protein calretinin, a marker for mossy cells (MCs), reveals that MCs constitute a substantial proportion of the PI‐labeled cells in the hilus. eYFP, enhanced yellow fluorescent protein; GCL, granule cell layer; h, hilus.

Previous in vivo studies have demonstrated that extended DG GC discharge induces hippocampal injury, with a selective loss of DG hilar neurons.^6^, ^23^, ^24^ We adapted the stimulation protocol from Kienzler et al.,^24^ taking into account the slower kinetics of ChR2 compared to electrical stimulation. A custom‐made stimulation device—comprising a stimulation box equipped with six high‐power 505‐nm LED modules and a Raspberry Pi‐controlled LED driver—was built to facilitate the stimulation of OTCs under standard culture conditions for an extended period of time (Figure 2A). After chronic optical stimulation for 6 h using the modified protocol (100 pulses with 505‐nm light, with 10‐ms pulse length, at a frequency of 12.5 Hz, repeated every minute), we assessed cell viability by incubating the OTCs with the DNA‐intercalating fluorescent dye PI. PI staining revealed substantial cell death, which was restricted to the DG hilar region, whereas the granular cell layer was unaffected and did not show any PI staining. Control OTCs, which were not transduced with ChR2, showed no signs of cell death after stimulation (Figure 2B). The severity of cell loss was dose‐dependent, and some cell loss was observed as early as 30 min poststimulation (Figure 2C). Immunofluorescent staining against the calcium‐binding protein calretinin, prevalent in mouse DG MCs,^25^ confirmed that most of the damaged hilar neurons were MCs (Figure 2D).

**FIGURE 3:**
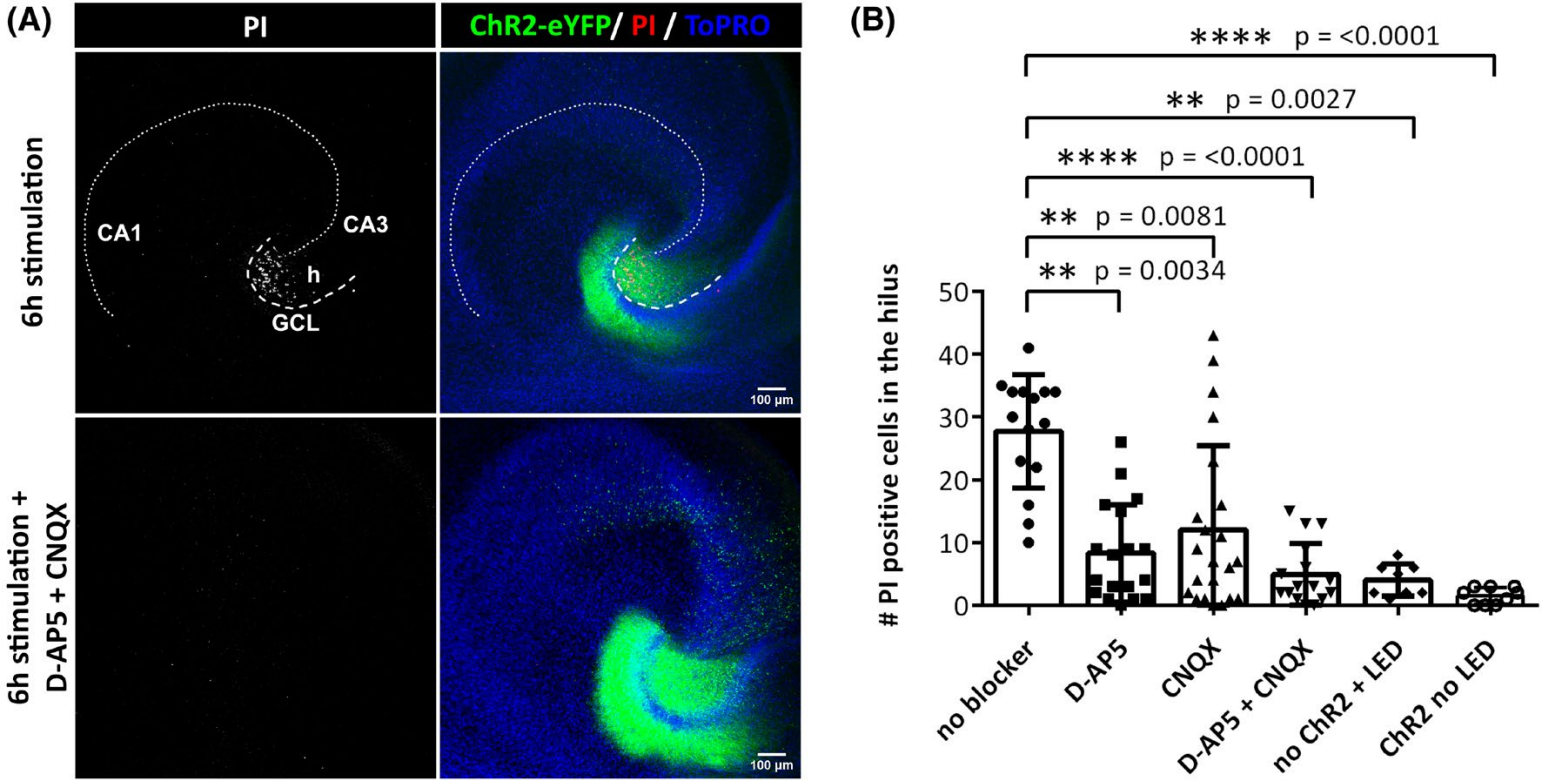
Inhibition of glutamatergic transmission prevents hilar neuronal cell death induced by chronic optical stimulation. (A) Dependence of hilar neuronal cell death on glutamatergic synaptic transmission was demonstrated through propidium iodide (PI) staining. Organotypic tissue cultures subjected to chronic optical stimulation while being treated with D‐2‐amino‐5‐phosphonopentanoic acid (D‐AP5) and 6‐cyano‐7‐nitroquinoxaline‐2,3‐dione (CNQX) exhibited minimal or absent PI‐labeling of cells in the hilus, indicating reduced cell death. (B) Quantification of PI‐positive cells in cultures treated with 10 μmol·L^−1^ D‐AP5 and 10 μmol·L^−1^ CNQX during a 6‐h optical stimulation revealed comparable numbers of PI‐positive cells in the hilus (mean = 4.9 ± 4.9) to baseline levels observed in channelrhodopsin‐2 (ChR2)‐negative cultures that were stimulated with the same stimulation protocol (mean = 4.0 ± 2.6). Differences across all groups were analyzed using the Kruskal–Wallis test with post hoc Dunn's multiple comparisons performed for pairwise comparisons; **p < .01, ****p < .0001 relative to stimulation without blockers; if not indicated in the graph, there was no significant difference between groups. *n* = 8–22 cultures per condition. Bars represent mean ± SD. eYFP, enhanced yellow fluorescent protein; GCL, granule cell layer; h, hilus; LED, light‐emitting diode.

To test whether the damage to MCs following optical stimulation was driven by excitatory synaptic input, we performed optical stimulation in the presence of α‐amino‐3‐hydroxy‐5‐methyl‐4‐isoxazolepropionic acid receptor and N‐methyl‐D‐aspartate receptor inhibition, via CNQX and D‐AP5, respectively. The inhibition of glutamatergic synaptic transmission effectively prevented hilar neuronal cell death (Figure 3), demonstrating that hilar neuron loss is synaptically driven.

### 3.3 Damage to hilar neurons via chronic optogenetic stimulation is attenuated by the anticonvulsant retigabine

**FIGURE 4:**
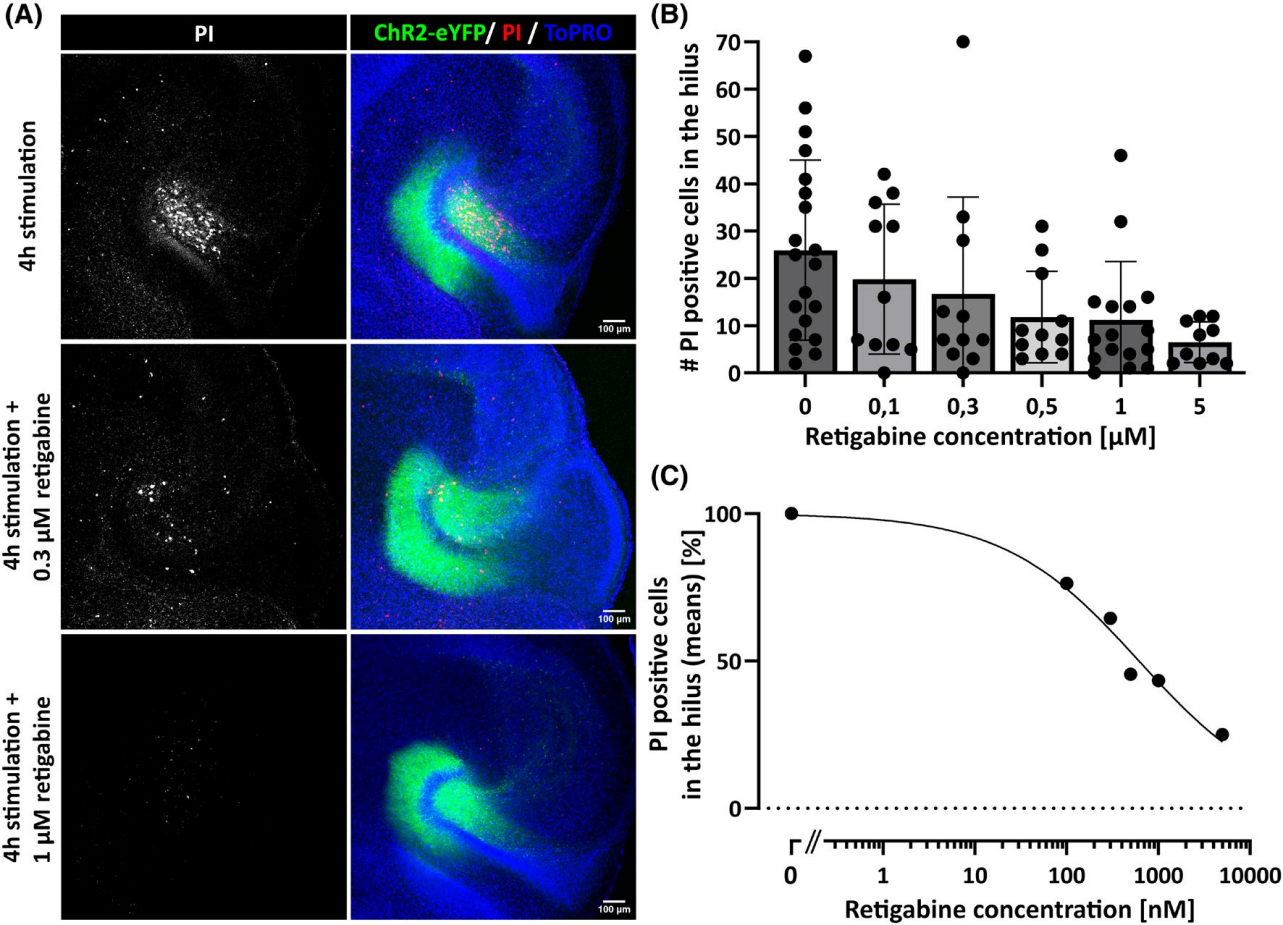
Retigabine protects hilar neurons from death following chronic optical stimulation. (A) Sample images of organotypic tissue cultures stained with propidium iodide (PI) after 4 h of optical stimulation. Treatment with retigabine during the optical stimulation prevented cell death, indicated by a reduction in PI staining. (B) The number of PI‐positive cells in the hilus was quantified with semiautomated cell counting. The addition of 5 μmol·L^−1^ retigabine to the culture medium reduced the number of PI‐positive cells by 75% (26.0 PI‐positive cells in the hilus ± 19.0 untreated vs. 6.5 ± 4.3 with 5 μmol·L^−1^ retigabine), indicating a protective effect of retigabine on hilar neurons against optical stimulation‐induced cell death. *n* = 10–20 cultures per condition. Bars represent mean ± SD. (C) The mean number of PI‐positive cells in the hilus for different retigabine concentrations was normalized to the untreated control and fitted with a nonlinear regression curve. Retigabine reduced the number of PI‐positive cells in the hilus in a dose‐dependent manner, with a median effective concentration of ~616 nmol·L^−1^. eYFP, enhanced yellow fluorescent protein; ChR2, channelrhodopsin‐2.

Due to the straightforward implementation of PI staining as an indicator of cell death, we aimed to investigate whether our in vitro optical stimulation model might also serve as a pharmacological tool for screening the neuroprotective potential of drugs. As a proof‐of‐concept experiment, we carried out the optical stimulation protocol (4 h, 100 pulses with 505‐nm light with 10‐ms pulse length at a frequency of 12.5 Hz, repeated every minute) in the presence of increasing concentrations of the anticonvulsant retigabine (Figure 4). Retigabine treatment resulted in a dose‐dependent decrease in the number of PI‐positive hilar neurons (Figure 4C).

### 3.4 Optogenetic stimulation of GCs elicits excitatory responses and induces degeneration in MCs

**FIGURE 5:**
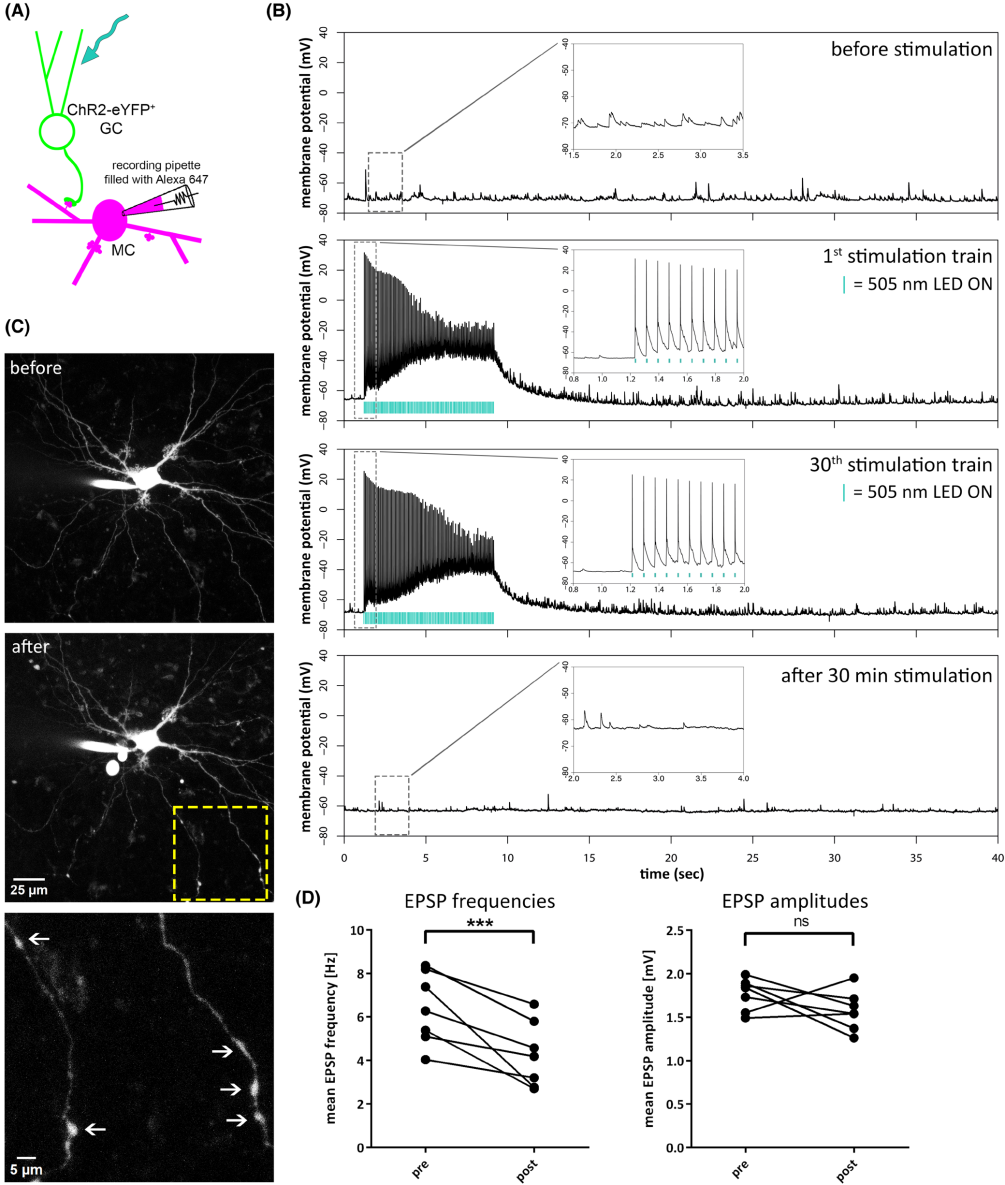
Repeated optogenetic stimulation of granule cells elicits excitatory responses and induces degeneration in mossy cells. (A) Schematic representation of the experimental procedure. Optogenetic stimulation of channelrhodopsin‐2 (ChR2)‐expressing granule cells (GCs) was performed using a 505‐nm light‐emitting diode (LED). Action potentials (APs) elicited in the GCs are transmitted via the mossy fibers to the mossy cells (MCs). Via whole‐cell patch‐clamp experiments, spontaneous and optically induced excitatory postsynaptic potentials (EPSPs) were recorded in identified hilar MCs. (B) Representative traces from a current‐clamp recording of an MC. During the initial 20‐min baseline period, spontaneous EPSPs were recorded. MCs exhibit a high frequency of EPSPs, with occasional large‐amplitude single events (10–21 mV). The same stimulation protocol used to induce hilar neuronal cell death in previous experiments was then applied (100 pulses with 10‐ms pulse length, at 12.5 Hz every minute). During the optical stimulation train, MCs displayed a pronounced depolarizing shift of the membrane potential, on top of which APs were generated. The stimulation was repeated 30 times, with a 52‐s intertrain interval. Following stimulation, spontaneous EPSPs were recorded for a maximum of 20 min. The first and last insets show a 2‐s time window of representative spontaneous activity. The second and third insets show the first 10 optically induced APs of the first and last stimulation train, respectively. Cyan lines indicate when the 505‐nm LED was switched on. (C) Confocal z‐stacks of the recorded MCs were captured at the beginning and end of the 70‐min recording period. After 30 min of optical stimulation, the MCs exhibited the first signs of degeneration, such as the formation of dendritic blebs (indicated by arrows). (D) Quantification of spontaneous EPSPs before and after stimulation within the same cell, showing a significant reduction in the mean EPSP frequency after optical stimulation (***p <.001, paired Brunner–Munzel test), whereas on average EPSP amplitudes stayed the same (ns *p* =.1406, paired Brunner–Munzel test). *n* = 7 cells. eYFP, enhanced yellow fluorescent protein; ns, not significant.

To study the effects of optogenetic GC stimulation on single MCs, we recorded spontaneous MC activity in a current‐clamp configuration for 20 min before and after a 30‐min optical stimulation (Figure 5A,B). This was prompted by our earlier observation using PI stainings of initial effects after only 30 min of stimulation (Figure 2C).

During the initial 20‐min baseline recording, MCs displayed a high frequency of EPSPs (6.4 ± 1.7 Hz, *n* = 7 cells). The mean EPSP amplitude was 1.76 ± .19 mV, and large‐amplitude EPSPs with amplitudes up to 21 mV were observed, reflecting strong mossy fiber input. During the optical stimulation train (100 pulses with 505‐nm light with 10‐ms pulse length at a frequency of 12.5 Hz), MCs exhibited tonically depolarized membrane potentials, on top of which APs were generated (Figure 5B). Poststimulation, the frequency of spontaneous EPSPs declined significantly (Figure 5D). Confocal z‐stacks of the recorded MCs were captured at the beginning and end of the 70‐min recording period. After the stimulation, the neurons exhibited initial signs of degeneration, such as the formation of dendritic blebs (Figure 5C).

## 4 DISCUSSION

This study aimed to define the temporal dynamics and mechanisms of activity‐dependent MC damage using an in vitro model with chronic optogenetic stimulation. Our key findings include (1) the successful transduction of GCs in OTCs with optogenetic constructs using AAVs, (2) selective damage to hilar neurons following optogenetic stimulation of GCs, (3) the ability of our model to evaluate the neuroprotective effects of drugs during stimulation, and (4) the identification of changes in EPSPs in hilar MCs after 30 min of optical stimulation.

### 4.1 MCs are synaptically activated by optogenetic stimulation of DG GCs

This OTC‐based in vitro model offers the advantages of a preserved, organotypic hippocampal network architecture. OTCs can be maintained over weeks or months, facilitating time‐lapse studies while providing easy accessibility for genetic manipulations, imaging, or electrophysiological recordings. We used optogenetics for noninvasive, chronic stimulation of GCs. We minimized phototoxic damage during the optical stimulation by using the ChR2(H134R) variant, which can be activated by lower light intensities,^22^ by employing 505‐nm light for stimulation and by adding a mixture of antioxidants^26^ to the culture medium prior to photostimulation. The viral transduction of GCs with ChR2(H134R)‐eYFP resulted in a high proportion of labeled GCs. Effective synaptic activation of MCs by optogenetically stimulated GCs was confirmed through whole‐cell patch‐clamp recordings. Despite not all GCs expressing ChR2‐eYFP, all recorded MCs showed robust light‐induced EPSPs, resulting in AP firing. This is likely due to the high convergence of mossy fiber input onto hilar MCs^4^ and highlights the potent synaptic activation of MCs by mossy fiber input.^27^

### 4.2 Specific damage to hilar neurons after chronic optical stimulation

PI staining revealed a consistent pattern of cell death in the hilus after chronic stimulation. To quantify PI‐positive cells and account for variability between cultures, we applied specific quality criteria before the analysis, including only cultures with a high level of ChR2‐eYFP expression. Moreover, normalization attempts based on hilar area volume did not yield significantly different outcomes for the analysis (data not shown).

Calretinin staining confirmed most PI‐positive cells to be MCs. However, we did observe calretinin‐positive cells that were not PI‐positive after 6 h of stimulation, indicating a population of surviving MCs. Furthermore, some PI‐positive cells were not stained by the calretinin antibody. Based on previous studies from Sloviter et al., these calretinin‐negative cells might be somatostatin‐expressing interneurons.^15^ It is worth noting that although calretinin is a reliable marker for MCs in the mouse hippocampus, its expression varies between the temporal and septal regions of the hippocampus. Moreover, there has been evidence for hilar γ‐aminobutyric acidergic interneurons expressing calretinin in mice.^25^

### 4.3 Increased vulnerability of MCs

Our findings confirm a high vulnerability of MCs to excitotoxicity, consistent with hippocampal sclerosis in TLE patients^8^, ^28^ and rodent models of prolonged excitation.^16^, ^23^ The susceptibility of MCs to excitotoxic cell damage is presumably the result of the release of glutamate during periods of elevated activity. It has been hypothesized that the strong convergence of mossy fiber input and the high fidelity of mossy fiber transmission likely contribute to the increased vulnerability of MCs.^10^ Furthermore, particularly large amounts of glutamate are released from individual giant mossy fiber boutons, which contain innumerable transmitter vesicles.^27^, ^29^ Thus, during periods of strong GC spiking activity, such as during seizures, excessive release of glutamate could cause MC degeneration, ultimately leading to hippocampal sclerosis.^6^ Our finding that ChR2 activation in GCs transsynaptically induced robust AP firing in all recorded MCs supports this hypothesis.

### 4.4 Synaptic dynamics and cellular integrity of MCs following optical stimulation

Here, we demonstrate the feasibility of employing chronic optogenetic stimulation to investigate the early processes of hippocampal injury. Whole‐cell patch‐clamp experiments revealed a decrease in the frequency of spontaneous EPSPs in MCs following optical stimulation of GCs. This reduction could be attributed to a suppression of spontaneous GC activity through increased inhibition. A strengthening of inhibitory synapses through repeated GC activity is also conceivable. We found consistent MC firing in response to almost every optical stimulus, which in turn could strongly activate inhibition in the DG. However, we cannot exclude that the observed decrease in EPSP frequency is caused by acute damage and degeneration of mossy fiber–MC synapses. We observed early signs of degeneration in some of the recorded MCs, such as dendritic beading, suggesting that degeneration of postsynaptic sites may contribute to the observed reduction in EPSP frequency. Similar dendritic swellings have been described on hilar MC and CA3 pyramidal neurons by Sloviter after extended perforant path stimulation.^23^ Meanwhile, GC degeneration seems to be an unlikely cause for the changes in EPSPs, as we did not observe GC damage in the PI stainings.

### 4.5 Advancing neuroprotective strategies: Possible new insights into MC survival from optogenetic stimulation in organotypic slice cultures

The techniques established in this study will serve as a foundation for future investigations, facilitating insights into MC degeneration and survival through integrated electrophysiological and morphological data. To date, clinical trials have failed to reduce seizure susceptibility through the preventative application of antiepileptic drugs after brain injury.^30^ However, initial tests show promise in evaluating the protective properties of treatments against excitotoxic damage with this in vitro model; we demonstrate this through the dose‐dependent preservation of hilar neurons during optical stimulation by the anticonvulsant retigabine.

Furthermore, although dissociated neuron cultures are useful for studying molecular mechanisms, they cannot assess differential effects on different cell types and network‐level interactions. Entorhino‐hippocampal OTCs retain the complex interplay of the various hippocampal neuron types,^31^, ^32^ thereby providing a more holistic view of the network dynamics following chronic stimulation. Moreover, compared to in vitro models investigating excitotoxic cell death by employing bath application of glutamate or kainic acid,^33^ our model offers a better understanding of synaptic dynamics, capturing possible modulations of the presynaptic compartment after stimulation. The optogenetic approach, through the selective expression of ChR2, allows for precise control over the origin and degree of neuronal stimulation. This precision enables the dissection of specific contributions of defined, singular episodes of increased synaptic MC activation, a level of detail unattainable in bath application models.

The model described in this study offers a valuable tool for monitoring the temporal sequence of hippocampal injury at a cellular level and subsequent changes in network activity, as it enables longitudinal approaches not feasible in vivo. This is particularly relevant as recent studies suggest that pathological conditions can cause a shift in the network effect of MC activity, with a strengthening of the GC–MC synapse.^11^, ^34^

MCs play a complex role in the context of epileptic seizures. Their degeneration is a hallmark of hippocampal sclerosis observed in patients with TLE.^28^ The question of whether the loss of MCs alone is sufficient to induce the development of a hyperexcitable hippocampal network remains a topic of debate.^13^, ^35^ Recent studies have demonstrated that MCs have a regulatory, seizure‐suppressing effect in the epileptic brain. MC activation reduced the seizure duration, whereas MC inhibition increased seizure severity.^1^, ^11^ Furthermore, recent findings suggest that the functional role of MCs may shift during pathological states, potentially mediated by synaptic reorganization and brain‐derived neurotrophic factor‐dependent mechanisms.^34^, ^36^ However, it is important to note that the model described in this report does not provide direct evidence regarding the initiation or propagation of seizures. Considering the multifactorial nature of epilepsy, no single model can adequately capture the entirety of its underlying processes and mechanisms. Here, we focus on the processes of excitotoxic cell damage to DG hilar neurons induced by chronic synaptic overstimulation. For a better understanding of the multifaceted processes underlying epileptogenesis, complementary studies are essential.

## 5 CONCLUSIONS

In conclusion, we successfully established a novel in vitro model using chronic optogenetic stimulation in OTCs. This approach promises to contribute to further advances in our understanding of the mechanisms underlying excitotoxic damage in hippocampal sclerosis, a key pathological feature in TLE. We demonstrate the selective vulnerability of hilar MCs to excitotoxic damage induced by GC stimulation. This model can now be used to evaluate the potential of candidate drugs against excitotoxic damage. It can also open new avenues for the exploration of the early stages of hippocampal injury and subsequent changes in network activity. It offers a valuable platform for future research investigating factors influencing MC degeneration and survival.

## AUTHOR CONTRIBUTIONS

Carolin Christina Koretz and Rebecca Schneider contributed to the data acquisition and analysis. Alexander Drakew, Jochen Roeper, and Thomas Deller participated in the experimental design and interpretation of data. Alexander Drakew contributed the custom‐written software used for data acquisition and analysis. Tassilo Jungenitz contributed materials and methods. Carolin Christina Koretz, Alexander Drakew, and Thomas Deller wrote the manuscript with contributions from all authors.

## CONFLICT OF INTEREST STATEMENT

T.D. has received honoraria from Novartis for lectures on human brain anatomy. All other authors declare that the research was conducted in the absence of any commercial or financial relationships that could be construed as a potential conflict of interest. We confirm that we have read the Journal's position on issues involved in ethical publication and affirm that this report is consistent with those guidelines.

## Supporting information


Appendix S1.

## Data Availability

The data that support the findings of this study are available from the corresponding author upon reasonable request.
