# Supplementary material for: Chronic optogenetic stimulation of dentate gyrus granule cells in mouse organotypic slice cultures synaptically drives mossy cell degeneration
Source: Epilepsia. 2025 Feb 12;66(5):1734–46. doi: 10.1111/epi.18314 (PMC12097467; doi:10.1111/epi.18314)
Supplement: Supplementary file 1 — Appendix S1. [file EPI-66-1734-s001.docx]

**Appendix S1, supplementary materials and methods**

***Organotypic tissue cultures, extended***

300 µm thick horizontal brain sections were cut on a vibratome (LeicaVT1200S) in dissection medium containing MEM with 25 mM HEPES, 0.1 mg/ml streptomycin, 100 U/ml penicillin, 0.45% glucose, and 2 mM glutamax (all purchased from Gibco Invitrogen) at 4°C. Subsequently, the hippocampal formation, including the adherent part of the entorhinal cortex, was dissected out under a stereo microscope (Stemi 2000, Zeiss) and positioned onto sterile membrane culture inserts (Millipore Millicell-CM, PICM ORG 50, 0.4 μm pore size, 30 mm diameter). The incubation medium contained 42% MEM, 25% Basal Medium Eagle, 25% heat-inactivated normal horse serum, 25 mM HEPES, 0.15% sodium bicarbonate, 0.65% glucose, 0.1 mg/ml streptomycin, 100 U/ml penicillin, 2 mM glutamax, adjusted to pH 7.30. The cultivation medium was replaced every 2–3 days. All tissue cultures were allowed to mature *in vitro* for at least 28 days in a humidified incubator (5% CO_2_, at 35˚C).

***Viral labeling, extended***

Membrane inserts with OTCs cultivated for 2–4 days were placed in a 30 mm petri dish lid containing pre-warmed (37°C) buffer, which consisted of 129 mM NaCl, 4 mM KCl, 1 mM MgCl2, 2 mM CaCl2, 4.2 mM glucose, 10 mM HEPES, 0.1 mM Trolox (Sigma-Aldrich), 0.1 mg/ml streptomycin, 100 U/ml penicillin, osmolarity adjusted to 380 mOsm/kg with sucrose (all reagents were obtained from Gibco Invitrogen unless otherwise noted), with a pH of 7.40. The petri dish was placed on the stage of an upright microscope (Nikon FN1) equipped with a camera and software (TrueChrome Metrics) and a 10x water immersion objective lens (NA 0.30, Nikon). The virus solution was filled into an injection pipette pulled from thin-walled borosilicate capillaries (Harvard Apparatus, GC150TF) with an approximate pipette resistance of 2-3 MΩ with a DMZ-Universal Electrode Puller (Zeitz). The pipettes were held by a microJECT pipette holder with a build-in valve (npi electronic), which was attached to a PDES pressure application system (PDES-DXH, npi electronic) for precise control of application pressure and time. To ensure a high density of labeled granule cells without spreading of the virus into the hilar area, four injections (single pulse, 80 mbar, 8 seconds) were applied along the dentate gyrus granule cell layer with approximately 50 – 100 µm distance apart into the suprapyramidal blade and crest.

***Optogenetic stimulation in the incubator, extended***

Six high-power LED modules (505 nm wavelength, LXML-PE01-0080, LUXEON Rebel LED) fitted with circular collimators are mounted onto an aluminum cooling base. The six-well plate with the OTCs was placed 2 cm above the LEDs so that every well of the well plate was positioned directly above one of the LED modules. The LED driver is controlled by a Raspberry Pi computer, allowing for precise control of all stimulation parameters via a custom-written Python script. LEDs were run at their maximum drive current of 1000 mA. The approximate light intensity was measured with a power meter (Thorlabs, PM160T) to be ~4.5 mW/mm^2^ at a distance of 2 cm from the LED. One stimulation train consisted of 100 light pulses with 10 ms pulse length at a frequency of 12.5 Hz. The inter-train interval was 52 seconds. OTCs were exposed to stimulation for increasing durations, ranging from 30 minutes to 6 hours. To protect cells from potential phototoxic damage by the high-intensity LED light, the following antioxidant reagents were added to the culture medium: 3.2 µM glutathione, 10 nM catalase, 110 µM ascorbic acid, 100 µM trolox, 2.3 µM α-tocopherol, 77 nM superoxide dismutase (all from Sigma-Aldrich).

***Whole-cell patch-clamp recordings, extended***

Whole-cell patch-clamp recordings of dentate gyrus granule cells and hilar mossy cells were performed 28 – 54 days post-viral transduction to ensure maturation of the mossy fiber synapses and allow for a high level of ChR2-eYFP expression. For recording, the membrane inserts with the cultures were transferred to a custom-made, 3D-printed recording chamber (made from MED-AMB, Rapidobject GmbH), where they were superfused with artificial cerebrospinal fluid (ACSF) containing 125 mM NaCl, 2.5 mM KCl, 1.25 mM NaH_2_PO_4_, 2 mM MgCl_2_, 2 mM CaCl_2_, 25 mM NaHCO_3_, 10 mM glucose, osmolarity adjusted to ~380 mOsm/kg with sucrose to match the incubation medium osmolarity and oxygenated with carbogen (95% O_2_ / 5% CO_2_). The temperature was kept at 35°C via the heating system of the recording chamber and heating sleeves for the objective and the condenser.

4–6 MΩ patch-pipettes were pulled from borosilicate glass (GC150TF-10, Harvard Apparatus) with a DMZ universal puller (Zeitz) and filled with K-gluconate-based internal solution: 135 mM K-gluconate, 5 mM KCl, 10 mM HEPES, 0.1 mM EGTA, 2 mM MgCl_2_, 2 mM MgATP, 0.2 mM Na_2_GTP, 10 mM PO-creatinine, and 0.3% (w/v) biocytin (Cayman Chemicals) pH 7.3, osmolarity adjusted to ~370 mOsm/kg with sucrose. 100 µM Alexa 647 dextran (Thermo Fisher) was added to the internal solution to immediately visualize the recorded cell.

Dentate gyrus granule cells and hilar mossy cells were visualized using infrared LED illumination (High Efficacy IR LED 850nm / 10W RSW-P10-850-0, Roschwege GmbH) and a Dodt-Gradient-Contrast system (Luigs & Neumann) mounted on a confocal laser scanning microscope (Fluoview 3000 on a BX63L upright microscope stand, equipped with a 60x NA 1.0 water immersion objective lens, Olympus/Evident). Cells were identified based on their somatic location: granule cells, characterized by small, round somata in the dentate gyrus granule cell layer, and mossy cells, distinguished by their larger somata in the hilus. To further confirm the identity of the recorded neurons, electrophysiological properties and cell morphology were used.

Spontaneous and optically induced activity of cells was recorded in current clamp mode using an ELC-03X amplifier (npi electronic) in bridge mode. Series resistance and pipette capacitance compensation were adjusted using a phase-sensitive technique (1). Amplifier output was low-pass Bessel-filtered at 20 kHz and digitized at 100 kHz using a PXI multifunctional I/O-module (NI PXI-6259 card in an NI PXI-1033 chassis, National Instruments) and in-house software based on LabVIEW (version 2023, National Instruments).

Spontaneous EPSPs were recorded for 20 minutes before and up to 20 minutes after optical stimulation. If cells depolarized permanently over -30 mV the recording was stopped before the 20-minute mark (3 out of 7 cells).

For optical stimulation, an LED module (505nm, LXML-PE01-0080, LUXEON Rebel LED) was mounted to a second micromanipulator opposite to the recording pipette. To ensure optimal illumination of the culture, the LED was positioned while measuring the stray light of the LED through the objective. Light pulses were delivered at trains of 100 pulses with 10 ms pulse length at a frequency of 12.5 Hz. For prolonged optical stimulation of mossy cells during patch-clamp recordings, trains were repeated over 30 minutes with an inter-train interval of 52 seconds.

For exact alignment of the electrophysiological traces with the optical stimulation, the potential output of the amplifier was wired to an FV3000 IO interface box (Evident/Olympus) and recorded as a current channel in the FV3000 software simultaneously with the PMT signal measuring the stray light of the stimulating LED through the objective.

***Automated analysis of spontaneous EPSPs, extended***

Traces were first denoised using a cubic spline fit, followed by a deconvolution (deconV(t) = V(t) + tau*dV(t)/dt) (2). This step effectively narrows the slow potential transients to sharp positive and negative peaks, thereby removing the pile-up of PSPs in time. The parameter tau is optimized in each trace to maximize the sharpening effect. We did not guess the PSP parameters directly from deconV(t) by reconvolution, as suggested by Richardson and Silberberg, because this turned out to be not robust enough in the present data set. Instead, the starting times of PSPs were obtained by the characteristic sequence of voltage steps making up the peaks in deconV(t). Then, V(t) was evaluated in sections between the starting times of sequential PSPs to extract the peak, the rising phase, and the decay of the PSP in that section. In cases where the preceding PSP had not fully decayed before the onset of the present PSP (pile-up), a guess of the further decay of that preceding PSP during the section under analysis was subtracted. In this study, the amplitude of the rise was considered for analysis.

**References**

1. Riedemann T, Polder HR, Sutor B. Determination and compensation of series resistances during whole-cell patch-clamp recordings using an active bridge circuit and the phase-sensitive technique. Pflugers Arch 2016; 468(10):1725–40.

2. Richardson MJE, Silberberg G. Measurement and analysis of postsynaptic potentials using a novel voltage-deconvolution method. J Neurophysiol 2008; 99(2):1020–31.
